# Hydronephrosis and renal failure following inadequate management of neuropathic bladder in a patient with spinal cord injury: Case report of a preventable complication

**DOI:** 10.1186/1754-9493-6-22

**Published:** 2012-09-26

**Authors:** Subramanian Vaidyanathan, Fahed Selmi, Kottarathil Abraham Abraham, Peter Hughes, Gurpreet Singh, Bakul Soni

**Affiliations:** 1 Regional Spinal Injuries Centre, Southport and Formby District General Hospital, Town Lane, Southport, PR8 6PN, United Kingdom; 2 Department of Renal Medicine, Southport and Formby District General Hospital, Town Lane, Southport, PR8 6PN, United Kingdom; 3 Department of Radiology, Southport and Formby District General Hospital, Town Lane, Southport, PR8 6PN, United Kingdom; 4 Department of Urology, Southport and Formby District General Hospital, Town Lane, Southport, PR8 6PN, United Kingdom

## Abstract

**Background:**

Condom catheters are indicated in spinal cord injury patients in whom intravesical pressures during storage and voiding are safe. Unmonitored use of penile sheath drainage can lead to serious complications.

**Case report:**

A 32-year old, male person, sustained complete paraplegia at T-11 level in 1985. He had been using condom catheter. Eleven years after sustaining spinal injury, intravenous urography showed no radio-opaque calculus, normal appearances of kidneys, ureters and bladder. Blood urea and Creatinine were within reference range. A year later, urodynamics revealed detrusor pressure of 100 cm water when detrusor contraction was initiated by suprapubic tapping. This patient was advised intermittent catheterisation and take anti-cholinergic drug orally; but, he wished to continue penile sheath drainage. Nine years later, this patient developed bilateral hydronephrosis and renal failure. Indwelling urethral catheter drainage was established. Five months later, ultrasound examination of urinary tract revealed normal kidneys with no evidence of hydronephrosis.

**Conclusion:**

Spinal cord injury patients with high intravesical pressure should not have penile sheath drainage as these patients are at risk for developing hydronephrosis and renal failure. Intermittent catheterisation along with antimuscarinic drug should be the preferred option for managing neuropathic bladder.

## Background

Hydronephrosis in persons with spinal cord injury is associated with the neural damage, urinary infection and back pressure; the importance of each factor varying considerably with the individual. [1] The appearance of the hydronephrosis associated with neurogenic bladder dysfunction has been attributed to the increased intravesical pressure which forces the ureter to pump urine into the bladder at increasingly higher pressures until a state of hydronephrosis is reached. [2]. Rosen and associates [3] recommend that all spinal cord injury patients functioning in the catheter-free state must be followed regularly at no longer than 6-month intervals. Changes in the resistance to the outflow of urine may occur at any time, even years after injury. When an event occurs, such as increased spasticity, which may be associated with increased sphincter resistance, the patient should be checked for evidence of outflow obstruction.

Condom catheters are convenient to spinal cord injury patients and their carers, but they can lead to problems and complications, sometimes severe. Newman and Price [4] found bacteriuria in more than 50% of patients using a condom catheter. Lesions of the penis can be secondary to mechanical damage to the skin from an excessively tight condom worn for a prolonged time. Another common cause of skin lesions is allergy to the material of the condom, usually to latex. Wyndaele and associates [5] concluded that a condom catheter may be indicated in male spinal cord injury patients with urinary incontinence provided that they have no penile lesion and that intravesical pressures during storage and voiding are urodynamically safe. We report a paraplegic patient, who had been managing his bladder by sheath drainage; twenty-five years after spinal cord injury, this patient developed bilateral hydronephrosis and renal failure. This case illustrates the perils of penile sheath drainage in spinal cord injury patients with high intravesical pressure.

### Case presentation

A 32-year old British white male person was struck on his back and head by a heavy pipe while working off-shore on a powerful drill in 1985.He had sustained fracture of T-12 vertebra with complete paraplegia at T-11 level. . This patient underwent fixation of lower thoracic spine with Harrington rods. A year later, there was displacement of distal Harrington rod hooks and slight bending of rods. Therefore, Harrington rods were removed. Immediately after spinal cord injury, this patient had indwelling urethral catheter. Two months after spinal injury, this patient was given Ubretid (Distigmine Bromide) intramuscular injection and was established on penile sheath drainage. Intravenous urography revealed well-defined pelvicalyceal systems, normal appearance of ureters, and urinary bladder with smooth outline. Eleven years after sustaining spinal injury, intravenous urography showed no radio-opaque calculus, normal appearances of kidneys, ureters and bladder.

Thirteen years after spinal injury, this patient developed recurrent urine infections. Intravenous urography revealed staghorn calculus in left kidney with no excretion of contrast at two hours; normal right pelvicalyceal system and ureter; normal bladder outline. MAG-3 renogram showed relative function of 21% in left kidney and 79% in right kidney. A JJ stent was inserted in left ureter and extracorporeal shock wave lithotripsy of left renal calculus was carried out. Biopsies of urinary bladder revealed follicular cystitis and marked cystitis glandularis. The urothelium contained occasional inflammatory cells but there was no evidence of dysplasia or malignancy. This patient was advised self-catheterisations.

Fourteen years after spinal injury, intravenous urography revealed no radio opaque calculi. Both kidneys excreted the contrast. Sixteen years after sustaining spinal injury, MAG-3 renogram revealed relative function of 13% by left kidney and 87% by right kidney. Blood urea: 6.2 mmol/L; Creatinine: 91 umol/L.

**Figure 1:**
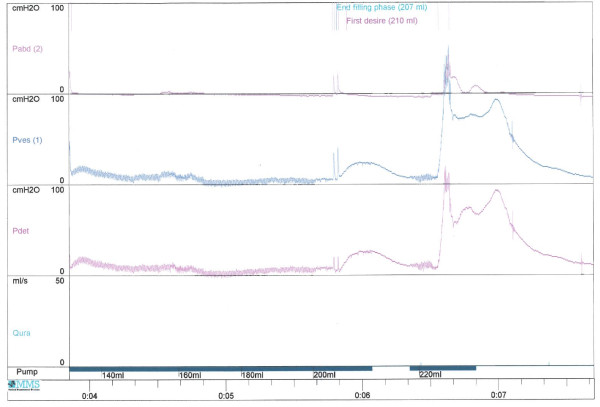
Urodynamics revealed that detrusor contraction was initiated by suprapubic tapping and detrusor pressure rose to 100 cm water.

Seventeen years after spinal cord injury, urodynamics revealed grade 1 vesicoureteric reflux on the right side through filling; no vesicoureteric reflux was seen on left side. Reflex detrusor contractions of 20–30 cm water. During these contractions, bladder neck opened and there was no exacerbation of vesicoureteric reflux. Detrusor contraction was initiated by suprapubic tapping and detrusor pressure rose to 100 cm water. (Figure 1) This patient was advised intermittent catheterisation with an anti-cholinergic drug, but this patient wished to continue as he was.

**Figure 2:**
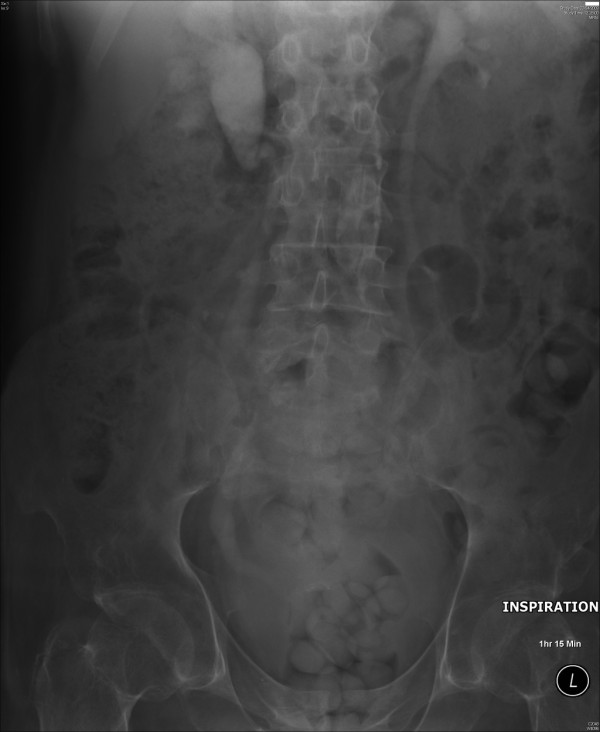
Intravenous urography – 75 minutes film showed both ureters and renal pelves filling progressively, which suggested a degree of raised pressure in the bladder.

Eighteen years after spinal injury, intravenous urography revealed staghorn calculus in the upper pole of left kidney. Percutaneous nephrolithotripsy of left renal calculi was performed. Twenty-four years after spinal injury, intravenous urography revealed a one mm diameter calcific density projected over the lower pole, and a five mm density at the upper pole of left kidney. There was right hydronephrosis with some hold up at the right pelviureteric junction but without actual obstruction. Contrast was seen throughout the left ureter. The lower right ureter was dilated; indeed both systems seemed to fill progressively throughout the examination suggesting a degree of raised pressure in the bladder. (Figure 2) No obstructing calculus was seen on either side.

**Figure 3:**
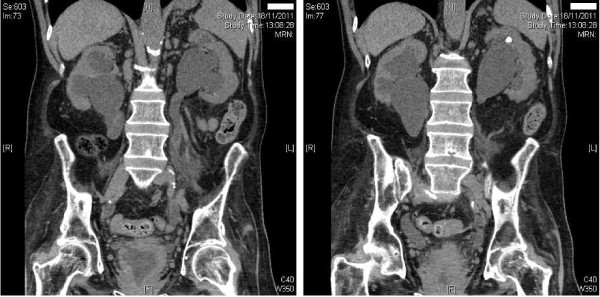
** A; (left panel) Computed tomography (coronal view) confirmed moderately severe bilateral hydronephrosis and hydroureter. B** (Right panel) Computed tomography (coronal view) revealed moderately severe bilateral hydronephrosis. A small calculus was present in the upper pole calyx of left kidney.

**Figure 4:**
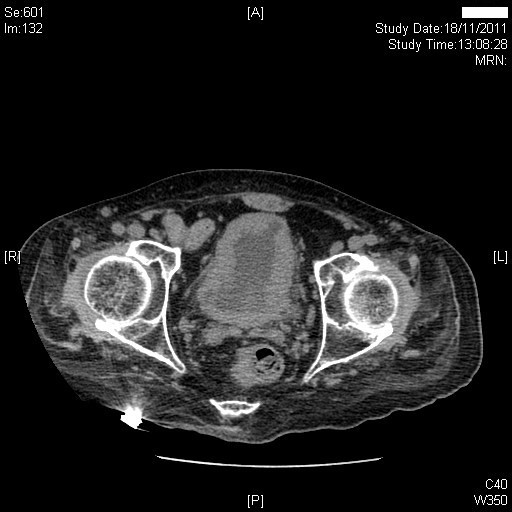
Computed tomography revealed marked circumferential thickening and trabeculation of the slightly contracted urinary bladder.

Twenty-six years after spinal cord injury, this patient developed shivering and stabbing pain in left kidney. Ultrasound scan revealed bilateral moderate to severe hydronephrosis with renal cortical thinning. Computed tomography confirmed bilateral moderately severe hydronephrosis and bilateral moderate hydroureters extending down to the vesicoureteric junction. (Figure 3 A and 3 B) A seven mm calculus in the upper pole of left kidney, 4 mm and 2 mm calculus in the lower pole of left kidney were seen, but there were no ureteric calculi. Bilateral renal cortical thinning (more marked on the right side) was present, which suggested that the bilateral hydronephrosis was not acute. There was marked circumferential thickening and trabeculation of the slightly contracted urinary bladder (Figure 4).

Blood tests revealed: Potassium: 5.6 mmol/L; Urea: 38.2 mmol/L; Creatinine: 418 umol/L; Calcium: 1.97 mmol/L; Phosphate: 1.92 mmol/L; Bicarbonate: 17 mmol/L. Gentamicin 160 mg was given intravenously. Urine sample, which was taken subsequent to administration of antibiotic, showed no growth.

Following discussion with the patient, indwelling urethral catheter drainage was established. This patient was prescribed Calcium resonium 15 grams four times a day with water, Sodium bicarbonate 1 gram three times a day after food, Calcium acetate 1 gram three times a day with meals. His condition improved remarkably. This patient was advised to take Trospium chloride 20 mg on alternate days.

**Figure 5:**
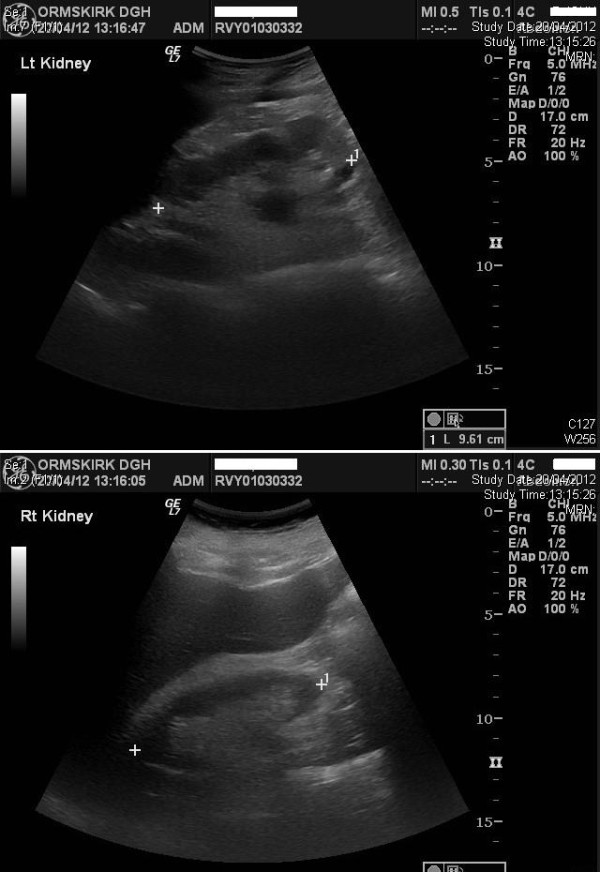
** A (Top panel): Ultrasound examination, performed five months after establishing indwelling urethral catheter drainage, revealed normal right kidney with no evidence of hydronephrosis. B** (Bottom panel) Ultrasound examination, performed five months after establishing indwelling urethral catheter drainage, revealed normal left kidney with no evidence of hydronephrosis.

Ultrasound examination was performed four weeks later; this revealed massive improvement in both kidneys with the previously distended collecting systems returning to almost normal appearances. Ultrasound examination of urinary tract was repeated after four months; both kidneys appeared normal in size and texture with no evidence of hydronephrosis. (Figures 5 A and 5 B) Blood tests revealed: Urea: 18.9 mmol/L; Creatinine: 197 umol/L; Calcium: 2.28 mmol/L; Phosphate: 1.28 mmol/L; Bicarbonate: 26 mmol/L. This patient was advised to perform intermittent catheterisations and get rid of indwelling urethral catheter.

## Discussion

Löfgren and Norrbrink [6] stated that in order to improve treatment outcome, health care professionals should listen to, respond to and respect the patient’s knowledge, experience and wishes. But some spinal cord injury patients may not be fully aware of the possible deleterious effects of unmonitored penile sheath drainage or long-term indwelling urinary catheter drainage. Our patient was informed that he could develop urine infections, worsening of vesicoureteric reflux and renal damage if he continued with sheath drainage. However, health professionals were unable to persuade this patient to perform intermittent catheterisation and take antimuscarinic drug. This case raises the importance of repeated discussions with the spinal cord injury patients to highlight the possibility of developing serious complications such as bilateral hydronephrosis and renal failure with unmonitored penile sheath drainage.

Price and associates [7] observed that the factors most frequently associated with renal deterioration were: (1) vesicoureteral reflux, (2) renal calculi, (3) recurrent pyelonephritis as demonstrated by calyceal blunting, and (4) recurrent decubitus ulcers, the latter usually in combination with other factors. Our patient had grade 1 vesicoureteric reflux on right side, left renal calculi and urine infections. This patient also had intravesical pressure exceeding 100 cm water when detrusor contraction was initiated by suprapubic tapping. A combination of high intravesical pressures, vesicoureteric reflux, renal calculi, and urine infections resulted in renal failure in this patient.

Kuo and Liu [8] administered OnabotulinumtoxinA 200 U detrusor injections to 33 patients with chronic spinal cord injuries; these injections were repeated every six months for four times. Patients were instructed to perform clean intermittent catheterization during the treatment and follow-up periods. Although mean bladder capacity increased from 207 to 412 ml and mean detrusor pressure decreased from 39.8 to 20.6 cm of water, the glomerular filtration rate decreased from 93.4 to 83.5 ml/min (P = 0.028). These authors were unable to demonstrate any improvement in glomerular filtration rate over a 24-month period in patients with chronic spinal cord injury, who received repeated detrusor injections of OnabotulinumtoxinA. We recommend intermittent catheterisation and antimuscarinic drugs by mouth as the preferred method of managing neuropathic bladder in spinal cord injury patients.

Prostate volume and Prostate Specific Antigen levels are lower in men with spinal cord injury and are inversely related to the patient’s age at the time of sustaining spinal cord injury. It is not clear as to whether this effect is mediated directly or indirectly by impaired nerve supply to the prostate. A study of 113 spinal cord injury patients (mean age 61.3 years) and 109 age-matched able bodied subjects (mean age 65.4 years) revealed significantly smaller prostate in spinal cord injury patients, as measured by ultrasound scan and digital rectal examination than those observed in able bodied subjects. Serum testosterone levels were lower in spinal cord injury patients when compared to able bodied subjects. [9] The subject of this case report sustained complete paraplegia as a result of spinal cord injury when he was 32 years old. Therefore, this patient has reduced likelihood of developing enlargement of prostate gland. However, occurrence of prostatic diseases (benign hypertrophy of prostate or carcinoma of prostate) should not be disregarded in spinal cord injury patients.

Good communication between healthcare professionals and patients is essential. National Institute for Health and Clinical Excellence (NICE), United Kingdom [10] recommends that the advice given to patients should be supported by *evidence-based written information* tailored to the patient’s needs. Treatment and care, and the information patients are given about it, should be culturally appropriate. It should also be accessible to people with additional needs such as physical, sensory or learning disabilities. We have started providing written information to spinal cord injury patients regarding possible complications of unmonitored penile sheath drainage and long-term indwelling catheter drainage (urethral as well as suprapubic catheter).

Hoffman and associates [11] from the Northwest Regional Spinal Cord Injury System, University of Washington, Seattle, WA, USA produced an in-person lecture and discussion series called the SCI Forum and videotaped it for on-demand viewing from their website. The Internet has the potential to provide user-friendly health information to people living with disabilities. Video media helps to enhance understanding and retention of health information compared to written or in-person instruction only. Thus advances in digital media and the Internet have made it possible to deliver more current, interesting, and useful information to people with spinal cord injury. In our spinal unit, we conduct inpatient education sessions. We try to publish in *open access journals* so that health professionals, carers and spinal cord injury patients can obtain information regarding management of spinal cord injury while browsing the internet.

## Conclusion

Condom catheters should be used only in those spinal cord injury patients in whom intravesical pressures during storage and voiding are safe. This case illustrates that penile sheath drainage in a spinal cord injury patient with high intravesical pressure can lead to bilateral hydronephrosis and renal failure.

### Consent

Written informed consent was obtained from the patient for publication of this Case report and accompanying images. A copy of the written consent is available for review by the Editor-in-Chief of this journal.

## Competing interests

The authors declared they have competing interest.

## Authors’ contributions

SV conceived the idea and wrote the manuscript. KAA provided renal support for this patient. PH reviewed radiological images. BMS and FS provided spinal care for this patient. GS and SV provided urological care to this patient. All authors contributed to and approved the final version of the manuscript.
